# Self and Body Part Localization in Virtual Reality: Comparing a Headset and a Large-Screen Immersive Display

**DOI:** 10.3389/frobt.2019.00033

**Published:** 2019-05-08

**Authors:** Albert H. van der Veer, Matthew R. Longo, Adrian J. T. Alsmith, Hong Yu Wong, Betty J. Mohler

**Affiliations:** ^1^Max Planck Institute for Biological Cybernetics, Tübingen, Germany; ^2^International Max Planck Research School for Cognitive and Systems Neuroscience, University of Tübingen, Tübingen, Germany; ^3^Department of Psychological Sciences, Birkbeck, University of London, London, United Kingdom; ^4^DEC, ENS, EHESS, CNRS, Institut Jean Nicod, PSL University, Paris, France; ^5^Werner Reichardt Centre for Integrative Neuroscience, University of Tübingen, Tübingen, Germany; ^6^Institute of Philosophy, Department of Philosophy and Media, University of Tübingen, Tübingen, Germany; ^7^Institute for Sport Science, Department of Human Sciences, Technical University of Darmstadt, Darmstadt, Germany; ^8^Max Planck Institute for Intelligent Systems, Tübingen, Germany

**Keywords:** self-consciousness, VR headset, multisensory cues, self-location, bodily self, large-screen immersive display, body part locations, body perception

## Abstract

It is currently not fully understood where people precisely locate themselves in their bodies, particularly in virtual reality. To investigate this, we asked participants to point directly at themselves and to several of their body parts with a virtual pointer, in two virtual reality (VR) setups, a VR headset and a large-screen immersive display (LSID). There was a difference in distance error in pointing to body parts depending on VR setup. Participants pointed relatively accurately to many of their body parts (i.e., eyes, nose, chin, shoulders, and waist). However, in both VR setups when pointing to the feet and the knees they pointed too low, and for the top of the head too high (to larger extents in the VR headset). Taking these distortions into account, the locations found for pointing to self were considered in terms of perceived bodies, based on where the participants had pointed to their body parts in the two VR setups. Pointing to self in terms of the perceived body was mostly to the face, the upper followed by the lower, as well as some to the torso regions. There was no significant overall effect of VR condition for pointing to self in terms of the perceived body (but there was a significant effect of VR if only the physical body (as measured) was considered). In a paper-and-pencil task outside of VR, performed by pointing on a picture of a simple body outline (body template task), participants pointed most to the upper torso. Possible explanations for the differences between pointing to self in the VR setups and the body template task are discussed. The main finding of this study is that the VR setup influences where people point to their body parts, but not to themselves, when perceived and not physical body parts are considered.

## Introduction

In recent years, virtual reality technology has been increasingly used for basic and clinical neuroscience and behavioral research, see e.g., reviews by Alsmith and Longo (2019), Bohil et al. (2011), Slater and Sanchez-Vives (2016), and Ehrsson (2012). VR technologies can vary significantly in terms of the visual and bodily cues available to users. Heydrich et al. (2013) directly compared headsets using video-generated vs. computer-generated visual information and discussed the potential differences these technologies introduce to the study of bodily self-consciousness, while other studies have also used large-screen immersive displays (LSIDs) to study body and space perception (Piryankova et al., 2013; Mölbert et al., 2017). Both Heydrich et al. (2013) as well as Piryankova et al. (2013), report underestimation of egocentric distances in VR headsets (also: Loomis and Knapp, 2003; Renner et al., 2013), although egocentric distance has been found to be underestimated less (under 20%) in the Oculus Rift headset, than in older VR headsets (up to 60%) (Young et al., 2014; Creem-Regehr et al., 2015). Piryankova et al. (2013) investigated distance estimation also in large screen displays and found underestimation to occur in three different LSIDs. For the panoramic LSID (Pano-LSID) also employed in the current study, Piryankova et al. (2013) found that the distance to the screen influenced distance estimates such that these distances were “pulled toward” the distance to the screen. Mohler et al. (2010) and Ries et al. (2008) demonstrated that experience with a self-animated avatar improves distance estimates in VR headsets, although the reason for this is not fully known.

### Self-Location

Generally, people locate themselves where their bodies are. Although counter-examples are known, involving e.g., self-perception from the third-person perspective (Galvan Debarba et al., 2017; Gorisse et al., 2017), autoscopic phenomena (Blanke et al., 2008), or full-body illusions (Lenggenhager et al., 2007), it is more typically the case that people would indicate their bodies as where they are. In the present study, we investigate a specification of this bodily self-location. By asking participants to point directly at themselves, we aim to determine whether there is a bodily location, or set of locations, in which people think of themselves as precisely located.

Most literature focusing on specifying self-location in the body has used an outline of a human body where the task did not involve pointing to oneself but rather localization of a person or on a depiction of a person. Limanowski and Hecht (2011) asked participants to indicate the “center of the self” by placing markers on human silhouettes and found a dominant role for the brain. They also found that most individuals seem to believe there is one single point inside the human body where their self is located. Anglin (2014) used open questions and forced-choice self-localizing on a body silhouette and found in contrast that some participants reported that the self is not centralized in one location. Overall, she found participants locating the self and mind in the head and the soul in the chest. Starmans and Bloom (2012) asked people to judge when objects were closer to a depicted person, as well as to erase as much as possible of a picture of a stick figure named Sally, while still leaving Sally in the picture (Starmans and Bloom, 2011). They suggested on the basis of their results that people locate the self mainly in the head and, more particularly, in or near the eyes. Van der Veer et al. (2018) found in a paper-and-pencil task of pointing to oneself on a body outline that people pointed primarily to the upper torso, followed by the upper face.

Alsmith and Longo (2014) developed a method for eliciting precise self-location judgments concerning one's own body, rather than a depiction of a body, which also allowed specification of multiple bodily locations across trials. They adapted a version of a task developed by Howard and Templeton (1966), originally designed for locating the point of projection of binocular vision. The task required the subject to manually align a visually presented rod along the horizontal plane such that the near end pointed “directly at himself.” Alsmith and Longo (2014) adapted this task, requiring subjects to either haptically or visually align a rod along a sagittal plane, with individual trials split equally between two directions of rotation (upwards or downwards). They found that participants' judgments were not spread out homogeneously across the entire body, nor were they localized in any single point. Specifically, pointing was mainly to the upper face and the upper torso. Van der Veer et al. (2018) extended Alsmith and Longo's paradigm in a VR headset and found almost exclusively pointing to the upper face, followed by a much smaller amount to the upper torso. Recently, Alsmith et al. (2017) employed a paradigm where self-location is implicated by the part(s) of the body used by participants to indicate the locations of external objects relative to themselves. Using this more implicit method, they found evidence for the use of a weighted combination of head and torso for self-location judgments. All these self-location paradigms, except Van der Veer et al. (2018), are performed without the use of VR technology.

### Body Part Localization

It has been assumed, that the somatosensory system has access to accurate information of one's body size and shape (Soechting, 1982; Van Beers et al., 1998). However, most individuals have to be taught to correctly draw human body proportions (Fairbanks and Fairbanks, 2005), otherwise their drawings demonstrate several systematic distortions (Kahill, 1984). Moreover, multiple studies have shown structural distortions in position sense in healthy populations, which may involve distortions in body representations (Hach and Schütz-Bosbach, 2010; Longo and Haggard, 2010, 2012; Fuentes et al., 2013; Linkenauger et al., 2015; Saulton et al., 2015; Longo, 2017). Comparable methods have been used in patient populations, finding distortions in body size perception [in anorexia nervosa (AN): Gardner and Brown (2014), in AN and bulimia nervosa: Mölbert et al. (2017)].

There exist several methodologies for measuring body part localization on the physical body. When testing patients' abilities to localize body parts, it is common to have them point to specific parts of their own or the examiner's body (Sirigu et al., 1991; Felician et al., 2003), or to objects placed on specific locations of their own body. The body part target instructions can be in one of a diversity of forms, e.g., spoken, written, pictorial, pointing, or touching (Felician et al., 2003). To test for patients' ability to *identify* body parts, Semenza and Goodglass (1985) used a variety of tasks involving pointing to and touching of one's own and a depicted body and body parts. Also, in the study of body representations, pointing to one's own body parts with one's own hand has been employed as a measure of body part locating ability (Paillard, 1999). In studies of personal or body space (the space that your physical body occupies), Hach and colleagues asked participants to point with their hand—with or without the help of a laser pointer—to several landmarks on their own physical bodies while their body except their face was hidden from view (Hach and Schütz-Bosbach, 2010), and to body parts on their own bodies imagined in front of oneself (Hach et al., 2011). Longo and colleagues (Longo and Haggard, 2010; Tamè et al., 2017) had participants indicate with a baton where they perceived specific spatial landmarks on their occluded hands. They found specific distortions relative to the physical hand, namely overestimation of hand width and underestimation of finger length. These paradigms rely on physical self-directed pointing with the finger or an apparatus, either to one's own (physical) body or on a plane occluding the body from vision. Fuentes et al. (2013) had participants provide estimates of body part locations on a non-co-located body in a desktop body image task (BIT). On a computer screen a head was seen which was to be imagined as a mirror image of yourself and several body parts were to be located relative to this head. For a review of the body representations and the types of information processing involved in pointing to body parts, as well as the disorders it is affected by, see De Vignemont (2010).

### The Current Study

Although VR has become a widely used research tool for studying multisensory body perception and self-consciousness (Bohil et al., 2011; Ehrsson, 2012; Blanke et al., 2015; Slater and Sanchez-Vives, 2016), the influence of VR technology on self or body part localization has not been thoroughly investigated. In the current study, we investigate self- and body part localization in two VR setups (Pano-LSID and a VR headset) with the intention of directly comparing the results. We ask participants to indicate their self-location and several of their body parts' locations by rotating a virtual pointing stick through their sagittal plane. One way of considering the possible presence of distortions in body part localization—both general ones and those related to visual perception in VR—is not to assume the physical body as the best baseline for determining where people point to themselves. Therefore, a measure of where participants locate their body parts in VR is assessed to take into account the possible effects of such distortions on the measure of self-location by allowing for the normalization of pointing to self with regard to participants “perceived” body part locations. Fuentes et al. (2013) and Linkenauger et al. (2015) are previous studies employing the rescaling of body shapes on the basis of experimentally found perceived body part locations. Following the VR experimental trials, we also perform a body template task as in Van der Veer et al. (2018).

We have the following three main research questions. (1) Does pointing to self and body parts differ between an LSID and a VR headset? (2) Is indicated self-location in the body template task outside of VR similar to self-localization in VR? (3) Where do people precisely locate themselves (point to themselves) in their bodies?

Connected to these questions we have the following three predictions. (1) We predict that differences between the VR setups (specifically, visual access to the body, presence of a headset, and differences in spatial perception) will result in differences in self- and body part localization between the two VR setups. (2) In contrast to the VR Setups, where we expect face followed by torso, we expect participants to mainly indicate the upper torso, followed by the upper face (Van der Veer et al., 2018) as self-location in the body template task. (3) Given the most relevant previous literature (Alsmith and Longo, 2014; Van der Veer et al., 2018), we expect that participants will primarily point to the face and possibly also the upper torso in VR for self-location and that, if distortions in body part localization are present, that self-location will also differ in terms of physical vs. perceived body regions.

## Methods

### Participants

Thirty healthy volunteers [18 female; age: M = 29.2, *SD* = 9.8, range: 19–60 years; 27 right-handed (assessed by self-report)], naïve to the purpose of the experiment, participated, all with normal, or corrected-to-normal vision (including stereo depth vision). The participants were recruited from the participant database of the Max Planck Institute for Biological Cybernetics in Tübingen, Germany. All participants gave written informed consent. Procedures were in accordance with the principles of the Declaration of Helsinki and approved by the Ethics Committee of the University Hospital Tübingen.

### Procedure

The experiment was completely run in either German (18 participants) or in English (12 participants). The participants read an information sheet and signed an informed consent form. They were tested for stereo depth vision (Stereo Optical Co., Inc., Chicago, IL). Then the experimenter measured the height of the *top of the* participant's *head* (cranial vertex; *Kopfspitze*), *eyes* (pupils; *Augen*), *chin* (gnathion; *Kin*), *shoulders* (acromion; *Schultern*), *hips* (where the circumference is largest; *Hüften*), (tip of the) *nose* (*Nase*), elbows (the most laterally protruding part of the bone; Ellbogen), *waist* (where the circumference of the lower torso is smallest; *Taille*), *knees* (top of the knee cap; *Knie*), and *feet* (where the foot borders on the ankle; *Füße*) with a wooden folding ruler taped to a wall. During the measurement of these heights, the participant was instructed explicitly where the respective body parts are exactly located on the body (specified in brackets after the names in the list above) and which names they would hear for them over the loudspeakers during the experiment (these names are in *italics* in the list above; the German names are added in *italics* in brackets; elbows were not used for pointing). In order to ensure exact locations were known to the participants, they were briefly tapped on all the locations where they were to point at, while again the names of the locations were mentioned before the pointing task began.

**Figure 1:**
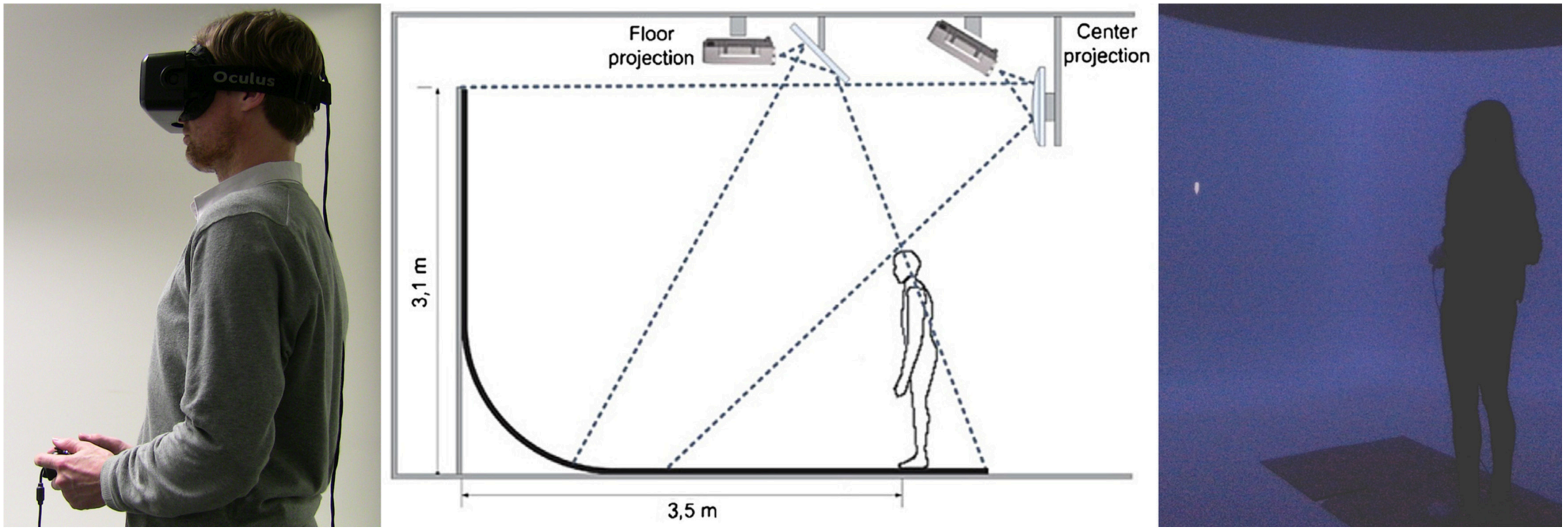
**(Left)** A photo of the VR headset experimental setup. The participant was standing still, wearing the VR headset, and holding the controller. The individual depicted has given written informed consent to appear identifiably in this publication. **(Middle)** A schematic depiction of the Pano-LSID experimental setup. Reused from Piryankova et al. (2013) with kind permission from the publisher (Elsevier). **(Right)** A photo of the Pano-LSID experimental setup. The participant was standing still in front of the Pano-LSID and holding the controller. In both setups the participant's task was to rotate a virtual pointer in their sagittal plane until they felt it was pointing “directly at you” or to a specific body part.

Participants were instructed that they would be asked to do a pointing to self-task in two VR setups: a VR headset (see Figure 1, Left) and a panoramic large-screen immersive display (Pano-LSID) (see Figure 1, Middle and Right). The order of the two VR setups was counter-balanced. After completing the self-pointing task, they were given instructions for a pointing to body part task (again counterbalanced in terms of VR setup). Following all VR pointing tasks the participants performed a body template task and two questionnaires.

#### VR Pointing Tasks

In the self-pointing task, the participants were asked: “[…] to adjust the direction in which the stick is pointing, so that it is *pointing directly at you*.” (or in German: “[…] die Richtung des Zeigestocks so zu verändern, dass dieser *genau auf Sie zeigt.”*). For the pointing to specific body parts task, the participants heard the previously instructed names of the body parts over the loudspeakers and were asked: “[…] to adjust the direction in which the stick is pointing, so that it is *pointing at different of your own body parts.”* (or in German: “[…] die Richtung des Zeigestocks so zu verändern, dass dieser *auf verschiedene Ihrer Körperteile zeigt*.”). For both pointing tasks, the participant used the joystick on the left-hand side of a controller to rotate the pointer upwards or downwards (both directions were permitted at all times) through their sagittal plane. They confirmed their preferred position by pressing a button on the right-hand side of the controller. Participants were asked to respond as accurately and quickly as possible and to stand still throughout the experiment.

Participants first completed the pointing to self-task in both VR-setups, then they completed the pointing to particular body parts in both VR-setups. Each time, before switching the VR setup, participants were allowed a short break where they were asked to sit down on a chair. Besides the breaks between the VR-setups, there was an extra break in the middle of each pointing at body parts block, where the participants could move a bit to stretch their legs. In the VR headset setup, the headset was kept on during these extra breaks.

#### Body Template Task

**Figure 2:**
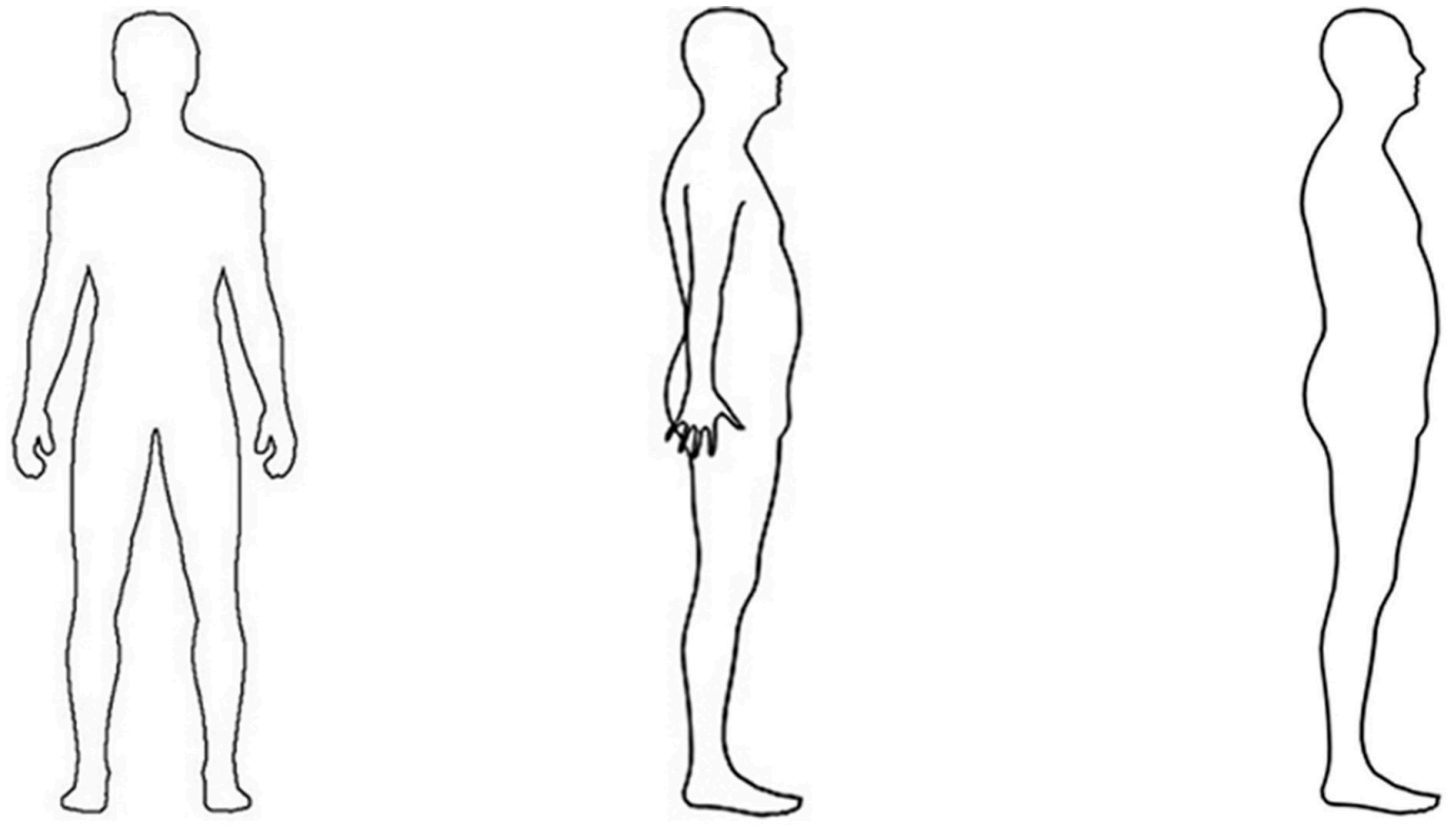
The body templates. The participant was asked to point “directly at you” on these three pictures of an outline of a body, under the assumption they were pictures of themselves.

After the VR pointing tasks were finished, participants performed a body template task, where they were asked to “Point directly at you” on an A4 print of a drawn body outline with a pen, under the assumption this was a picture of themselves. Participants performed this task on one frontal picture and on two side-view pictures, one with and one without an arm depicted (see Figure 2). The pictures were administered in counterbalanced order, with the frontal one always being the second. Based on our previous findings (Van der Veer et al., 2018), we predicted that participants in the body template task would point mainly toward the upper torso and to a lesser extent to the upper face.

#### Questionnaires

After the VR and the body template tasks, the awareness scale of the Body Perception Questionnaire (BPQ; self-measure, 45 items, five-option Likert scales) was administered (Porges, 1993). This questionnaire was included to test for possible correlations between task performances and a subjective self-report measure probing perceived interoceptive aptitude or interoceptive sensibility (for a discussion of these and other measures of interoception, see Garfinkel et al. (2015), where the term interoceptive *awareness* is reserved for the metacognitive level of the relationship between interoceptive accuracy and the awareness of this accuracy). Our interest in this questionnaire was to test if higher interoceptive sensibility correlates with more accurate body part localization. Correlations between interoceptive sensibility and pointing to self in the VR headset, the Pano-LSID, as well as on the body templates, were also tested.

Finally, a post-questionnaire (added to this article as a Supplementary Material) was filled out by the participant, with several questions about employed strategy.

### Experimental Setup

#### VR Headset

During the VR headset blocks (see Figure 1, Left), the participant stood in front of a table on which a Dell Precision M6700 laptop was positioned running the experiment. The computer had an Intel Core i7-3940XM central processor running at 3.00 GHz and an NVIDIA Quadro K5000M graphics card. An Oculus Rift development kit 2 VR headset with a diagonal field of view (FOV) of 96°, a resolution of 1920 × 1080 pixels (960 × 1080 per eye), and a frame rate of 60–75 Hz was used for stimulus presentation. The tracking camera of the Oculus Rift was mounted on a separate stand behind the table.

#### Pano-LSID

During the Pano-LSID blocks (see Figure 1, Middle and Right), the participant stood in front of a quarter-spherical panoramic large-screen immersive display with a radius of 3.5 m, a horizontal FOV of 230° (±115°) and a vertical FOV of 125° (25° upwards and 100° downwards onto the floor, up to 1 m behind the participant). Participants stood 3.5 m from the vertical screen (in all directions). The projection was done by six Eyevis LED DLP (ESP-LWXT-0.5) projectors, set up with 5 front projectors in portrait mode (1200 vertical × ~4500 horizontal pixels, 60 HZ) and 1 floor projector in landscape mode. Image rendering and warping and blending (performed through NVIDIA, GPU core) was done on a high-end cluster system consisting of seven computers, one client image generation PC for each projector plus a master PC where the experiment was run and the data recording was coordinated. All PCs were HP Z800 Workstations running at 3.47 GHz with ZOTAC nVIDIA GeForce 9800 GT graphics cards.

#### Both Setups

All the experimental blocks were run with lights out in the same room. The participant held a Microsoft Xbox 360 controller, moved the pointer using a joystick with the left hand and confirmed the decision by pressing a button with the right hand. Maximum speed of the pointer was 75°/s for the VR headset and 60°/s for the Pano-LSID, with the difference resulting from the different refresh rates of the setups.

### VR Stimuli and Experimental Design

The experiment was designed in Unity 4.6.7f1 for the VR headset and in version 4.2.1f4 for the Pano-LSID, employing the same code, resulting in completely analogous versions of the experiment in the two setups.

**Figure 3:**
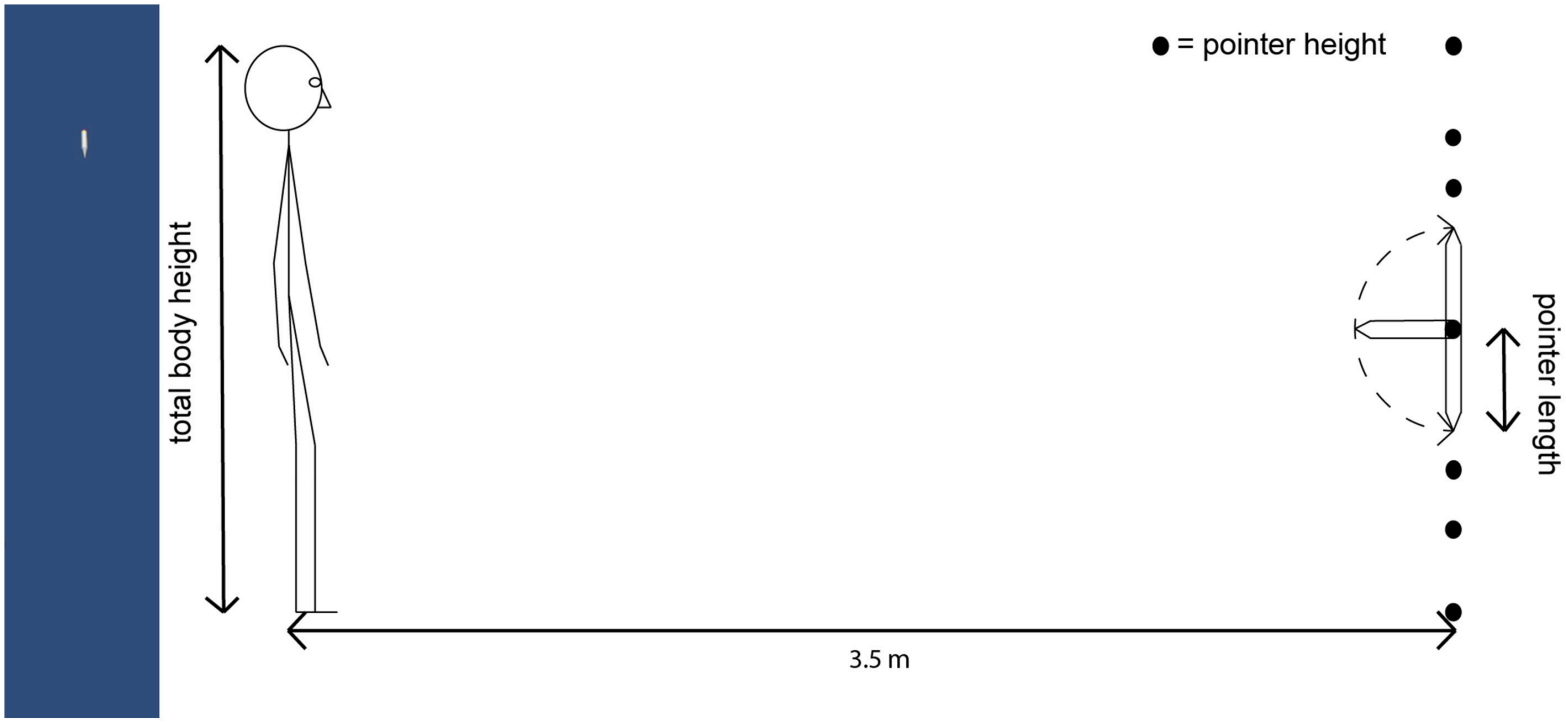
**(Left)** The pointer stimulus. An example image of the pointer stimulus, here with an angle of + 48.2° up from straight down, showing a field of view of about 20° horizontal and 100° vertical. **(Right)** Schematic depiction of the setup. The dotted line indicates the range of possible pointer rotations. The pointer starting direction was either straight up, straight down, or perpendicular to the participant. Seven pointer heights were spread out across the complete height of the participant's body: 0, 0.25, 0.5, 0.75, and 1 × total body height, middle between shoulder and chin height, middle between ground level and knee height.

The virtual environment consisted of an empty space with a blue background. In each trial the participant saw a cylindrical pointing stick with a blunt backside and a pointy front side. The backside of the pointer was fixed to a (non-visible) vertical plane orthogonal to the participant's viewing direction at 3.5 m distance from the participant (the distance to the vertical screen in the Pano-LSID). The pointer had a virtual length of 30 cm and a diameter of 4 cm, was a light-gray color, and had a fixed lighting source straight above, providing some shadow at the underside of the pointer (see Figure 3, Left). The starting direction of the pointer was pointing straight up, straight down, or perpendicular to the participant, at one of seven fixed backside heights: 0, 0.25, 0.5, 0.75, and 1 × total body height, the middle between shoulder and chin height, and the middle between ground level and knee height (see schematic in Figure 3). These different pointer starting directions and heights were included to make the task more diverse and to not cue participants, which might result from a more specific selection of angles and heights. The pointer starting direction perpendicular to the participant was also specifically added to test whether for the pointer height at the middle of the neck, i.e., between two regions of interest, the face and the torso, participants would choose to move the pointer substantially more up or more down to point at their self-location.

The complete experiment had a within-subject design with four factors: 2 × VR setup, 3 × pointer starting direction, 7 × pointer height, and 10 × target (self and 9 body parts), and one measure: pointing height (where an extension of the pointer at the chosen angle would intersect with the front of the participant's body). The number of trials was 3 (pointer starting directions) × 7 (pointer heights) = 21 per target for each VR setup, making 2 × 10 × 21 = 420 trials in total per participant. These trials took approximately 60 min to complete.

### Analysis

The measure recorded during the experiment was the angle of the pointer with the virtual plane to which its backside was fixed (with a range from 0° for completely down and 180° for completely up), when the participant indicated that the pointer was pointing “directly at you” or to a particular body part. Using the individualized height of the pointer, this angle was recomputed into the height where the virtual extension of the pointer would intersect with the front of the participant's body (the front of the body was taken as the virtual plane orthogonal to the participant's viewing direction, extending from the location of his eyes). All statistical analyses were performed in SPSS.

#### VR Self-Location on the Physical Body

**Figure 4:**
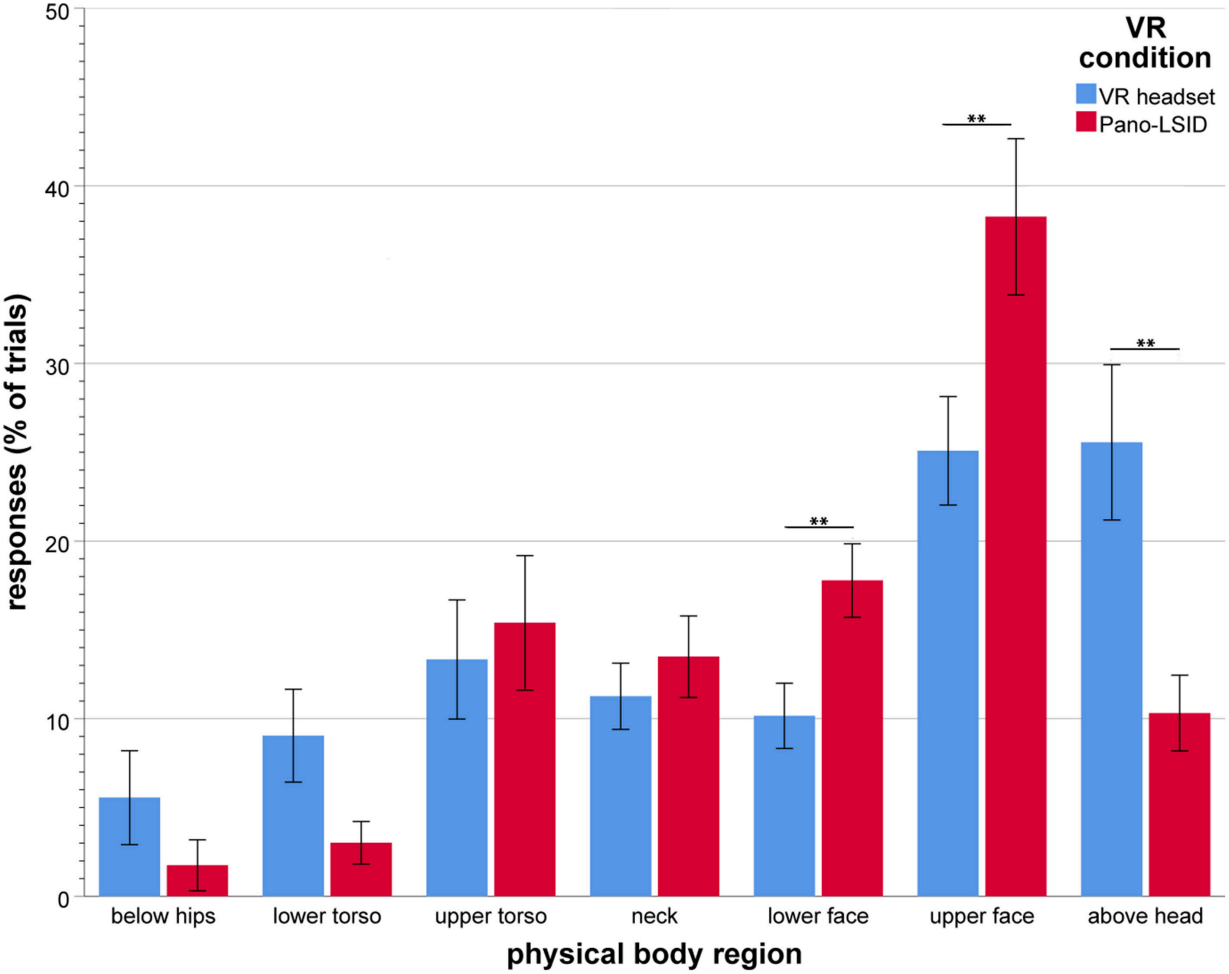
Self-pointing in terms of physical body regions (in mean percentage of trials per VR setup), by VR setup (*N* = 30; error bars: ± 1 SE; ^**^p < 0.01).

For self-pointing using the participant's individual body height measurements the height on the body was then classified as a score for one of seven regions of the body (in Figures 4, **8** these responses are shown in terms of percentages of trials per body region). As in earlier studies (Alsmith and Longo, 2014; Van der Veer et al., 2018), each response was coded as falling into a bodily region, depending on where it would intersect the body: below the torso (= below the hips), lower torso (= between the hips and the elbows), upper torso (= between the elbows and the shoulders), neck (= between the shoulders and the chin), lower face (= between the chin and the nose), upper face [ = between the nose and the top of the head (= total body height)], and above the head (= above total body height). These regions were chosen according to visually salient boundaries to facilitate coding, which correspond roughly to nameable body parts; head and torso are both split into two roughly equal regions, with a region between them, the neck, bounded by chin, and shoulders. The responses were analyzed using a RM-ANOVA, with factors body region (7 levels) and VR setup (2 levels). In case of (a) significant effect(s), relevant *t*-tests (corrected for false positives) were performed to further localize the effect.

For the trials with the pointer starting straight ahead and at the height of the neck, the percentages of trials pointed to the neck, to regions below the neck and to regions above the neck were compared; as well as the percentages of trials pointed down relative to the straight-ahead starting direction, up relative to this starting direction, and with no movement of the pointer.

#### VR Body Part Localization

For pointing at body parts, the pointing heights on the body were compared to the heights of the respective target body parts, as measured on the physical body, and the difference was taken as the measure error distance, in signed number of cm (with negative values being down and positive values up, relative to the physical height of the respective body part). The error distances were analyzed using a RM-ANOVA, with factors VR setup (2 levels) and target body part (9 levels). In case of (a) significant effect(s), relevant *t*-tests (corrected for false positives) were performed to further localize the effect.

The locations pointed at for the various body parts were subsequently used to rescale the average body across the sample, now based on the perceived body part locations instead of the physical body part locations, separately for the VR headset and the Pano-LSID. Pictures of the outline of the average body across the sample were made for the physical body, the body as perceived in the VR headset and the body as perceived in the Pano-LSID.

#### VR Self-Location on the Perceived Body

The *pointed at* body part locations were then used to recompute the regions of the body (see section VR Body Part Localization) used to categorize the height on the body for self-pointing. For each participant separately, these regions were recoded into new *perceived* body regions based on the *pointed at* body part locations (in **Figures 7**, **8** these responses are shown in terms of percentages of trials per body region), instead of on the *physical* body part locations. Subsequent recategorizing the self-pointing responses—considering where participants pointed out their body parts to be in the two VR setups—likely better reflects where they actually *experienced* themselves to be. Using the recategorized responses, i.e., numbers/percentages of trials scored per perceived body region, the RM-ANOVA was redone, to see whether the self-pointing for the two VR setups were different in terms of perceived body regions. In case of (a) significant effect(s), relevant *t*-tests (corrected for false positives) were performed to further localize the effect.

#### Body Template Task

Paired-samples *t*-tests were performed to test for differences between the pointing heights for the different body outlines used in the template task. It was also tested whether a significant correlation was present between the pointing heights for self in either VR setup (in percentages of total physical body heights) and on the body template (in percentages of total template body heights).

#### Questionnaires

The total score on the BPQ awareness scale was computed, as the mean score over all 45 items (scored from 1 to 5 each), with a higher score reflecting higher sensibility. It was tested whether a significant correlation was present between BPQ awareness score and absolute error distance for pointing to body parts (a negative correlation was hypothesized), or the pointing height on the body (as a percentage of total physical body height) for self-location in the VR headset, the Pano-LSID, or on the body template (all two-tailed Pearson correlations).

## Results

### VR Self-Location on the Physical Body

All results reported here are Greenhouse-Geisser corrected, because of failed Mauchly's tests of sphericity. There was a significant main effect of body region [*F*_(3.30, 95.7)_ = 12.0, *p* < 0.001, $\eta_{\text{p}}^{2}$ = 0.29; see Figure 4 for the responses per body region by VR condition]. Participants did not point to all regions of the body equally, nor did they point to one particular region only. They pointed mainly to the upper face (M = 31.7%, *SD* = 18.01), above the head (M = 17.9%, *SD* = 15.34), the upper torso (M = 14.4%, *SD* = 17.53), the lower face (M = 14.0%, *SD* = 9.31), as well as to the neck (M = 12.4%, *SD* = 10.41), with the upper face as the region pointed to most.

This effect of body region was modulated by a significant interaction between region and VR setup [*F*_(3.57, 103)_ = 9.32, *p* < 0.001, η${}_{\text{p}}^{2}$ = 0.24]. Simple main effects of body region were found to be significant for each VR condition separately [VR headset: *F*_(6, 24)_ = 4.03, *p* = 0.006, η${}_{\text{p}}^{2}$ = 0.50; Pano-LSID: *F*_(6, 24)_ = 27.4, *p* < 0.001, η${}_{\text{p}}^{2}$ = 0.87].

Specific comparisons were made between the VR conditions for each body region (7 comparisons) using the Holm-Bonferroni correction procedure. Holm-Bonferroni corrected paired-samples *t*-tests showed significantly less pointing for the VR headset compared to the Pano-LSID for the lower face (*p* = 0.0017) and the upper face (*p* = 0.0071), while more pointing was found above the head in the VR headset (*p* = 0.0050) (see Figure 4).

For the pointer at the height in the middle between shoulders and chin (i.e., the middle of the neck) and starting straight ahead, pointing was mostly to the neck (36.7% for both setups), followed by upper and lower face [VR headset: upper face (30.0%), then lower face (13.3%); Pano-LSID: lower face (36.7%), then upper face (23.3%), and little pointing to the upper torso (VR headset: 13.3%, Pano-LSID: 3.3%)]. In terms of directions, pointing was mostly up from the starting direction (VR headset: 60.0%; Pano-LSID: 60.0%), followed by no pointer movement (VR headset: 16.7%; Pano-LSID: 30.0%) and down from the starting direction (VR headset: 23.3%, Pano-LSID: 10%). So, in terms of directions participants showed a clear preference for going up rather than down when the pointer was starting out straight ahead at the neck.

### VR Body Part Localization

Five extreme outlier responses (from 4 participants; out of a total of 11,340 trials), which were the result of the task not having been correctly performed for the respective trials (the pointer was left in the starting direction, either straight up or straight down), were removed and values of 0 were imputed.

**Figure 5:**
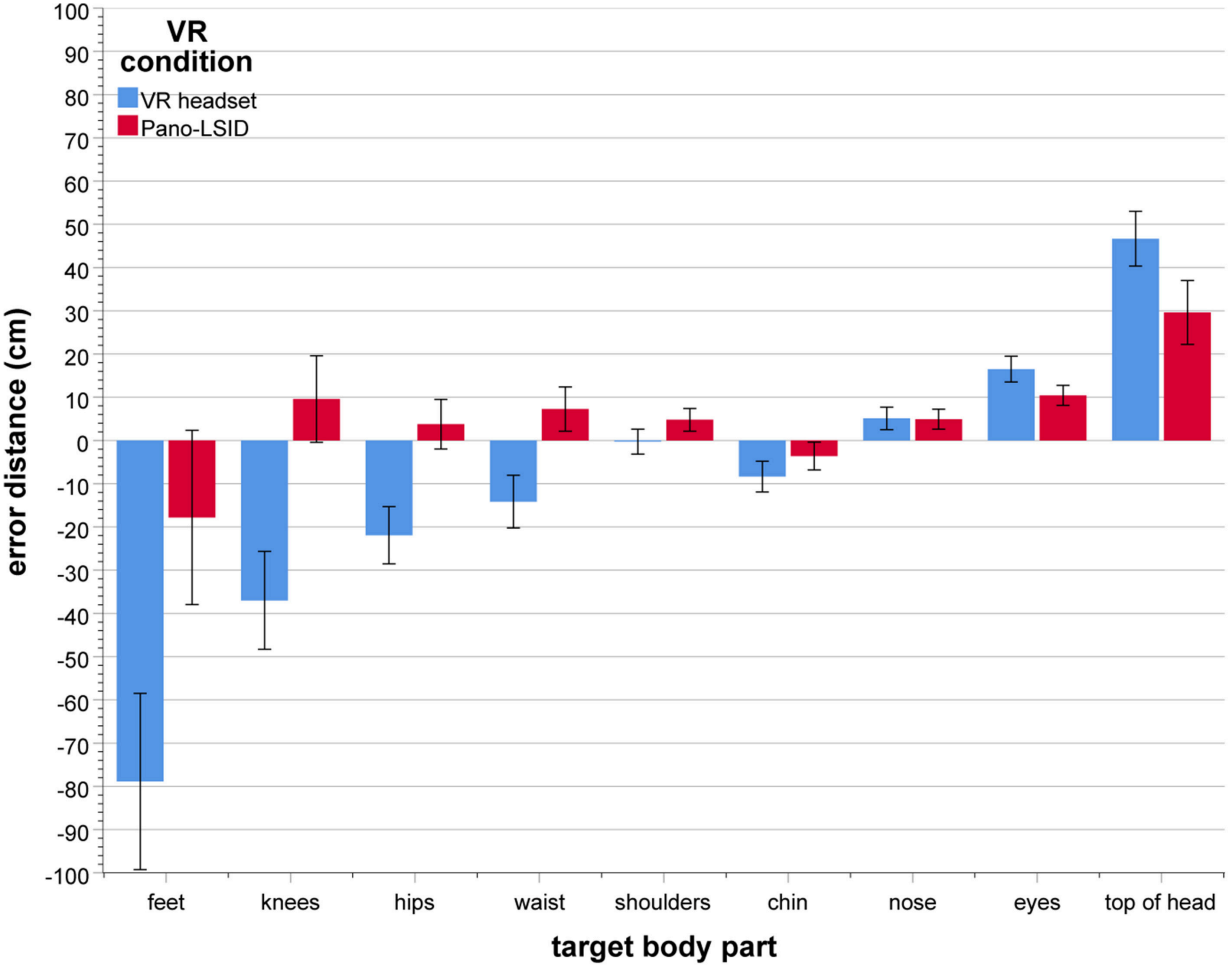
Mean error distance (in cm) between pointed at and physical body part location, per target body part, by VR setup (*N* = 30; error bars: ± 1 SE). The error distances are directional, with negative being down and positive being up relative to the physical height of the target body part per participant.

Where Mauchley's test indicated violation of the sphericity assumption, the Greenhouse-Geisser correction was applied. Significant main effects were found for target [*F*_(1.23, 35.7)_ = 9.57, *p* = 0.002, η${}_{\text{p}}^{2}$ = 0.25), as well as for VR setup [*F*_(1, 29)_ = 91.3, *p* < 0.001, η${}_{\text{p}}^{2}$ = 0.76] (see Figure 5 for the error distances per target by VR condition). The following trend for target is observed: from large undershooting for the lowest to large overshooting for the highest target (feet: M = −48.32 cm, *SD* = 108.61; knees: −13.69 cm, *SD* = 56.61; hips: −9.10 cm, *SD* = 33.09; waist: −3.45 cm, *SD* = 29.80; shoulders: 2.25 cm, *SD* = 14.55; chin: −5.97 cm, *SD* = 17.87; nose: 5.01 cm, *SD* = 12.25; eyes: 12.46 cm, *SD* = 13.63; top of head: 38.15 cm, *SD* = 34.11). For VR condition the following was observed: large mean undershooting for the VR headset (M = −10.22 cm, *SD* = 25.80) and small mean overshooting for the Pano-LSID (M = 5.44 cm, *SD* = 25.0).

The two main effects of target and VR condition were modulated by an interaction between these two factors [*F*_(2.23, 64.7)_ = 37.4, *p* < 0.001, η${}_{\text{p}}^{2}$ = 0.56; see Figure 5]. Considering the mean error distances, participants were able to point reasonably accurately (with mean error distances of around ±10 cm) to most of their body parts in the Pano-LSID, but much less so in the VR headset. In the VR headset mean pointing height to the top of the head was overestimated (M = 46.67 cm, *SD* = 34.58) and mean pointing to the feet (M = −78.87 cm, *SD* = 111.57), knees (M = −36.98 cm, *SD* = 61.99), and hips (M = −21.92 cm, *SD* = 36.17) were underestimated. In the Pano-LSID mean pointing height to the top of the head was overestimated (M = 29.63 cm, *SD* = 44.45) and pointing to the feet was underestimated (M = −17.81 cm, *SD* = 110.42). Inconsistency in the pointing—as indicated by large standard errors—can be observed particularly for the top of the head, the feet, and the knees, for both setups.

Simple main effects of target were found to be significant for each of the two VR setups separately [VR headset: *F*_(8, 22)_ = 5.16, *p* = 0.001, η${}_{\text{p}}^{2}$ = 0.65; Pano-LSID: *F*_(8, 22)_ = 9.74, *p* < 0.001, η${}_{\text{p}}^{2}$ = 0.78].

Specific comparisons were made between the VR conditions for each body part (9 comparisons) using the Holm-Bonferroni correction procedure. Holm-Bonferroni corrected paired-samples *t*-tests showed significant differences between the mean error distances for the VR headset and the Pano-LSID for all body parts, except the nose. The mean negative error distance for the feet was significantly larger for the VR headset compared to the Pano-LSID (*p* < 0.001). There was a significant difference between the negative mean error distance for the VR headset and the positive mean error distance for the Pano-LSID for the knees (*p* < 0.001), hips (*p* < 0.001), waist (*p* < 0.001), shoulders (*p* = 0.0053), and chin (*p* = 0.035). Finally, the mean positive error distance was significantly larger for the VR headset compared to the Pano-LSID for the eyes (*p* = 0.015) and the top of the head (*p* = 0.019).

There is some intrinsic mathematical bias in how the pointing heights on the body are derived. The mapping from angle to projected height on the body is not fully linear, but promotes a skewed distribution. This bias is not yet present at the level of the original pointing angles, so averaging over pointing angles first, before mapping onto pointing heights on the body, (partially) minimizes this bias. Therefore, new pointing heights were computed based on pointing angles averaged over several trials (of the same participant, target, VR condition, and pointer height). Statistical analyses were (re)run separately for the new and for the previous pointing heights (computed from pointing angles on a trial-by-trial basis), for body parts and for self. Comparing the results for the new and the previous pointing heights revealed only slight differences in the test statistics and no differences with respect to significant effects, for both body parts and self. The results from performing the alternative analyses of our data, partially reducing the effect of the mapping bias, do not call for a change in the interpretation of the data or our conclusions. We believe, it is therefore warranted to analyze the body heights computed from pointing angles on a trial-by-trial basis.

**Figure 6:**
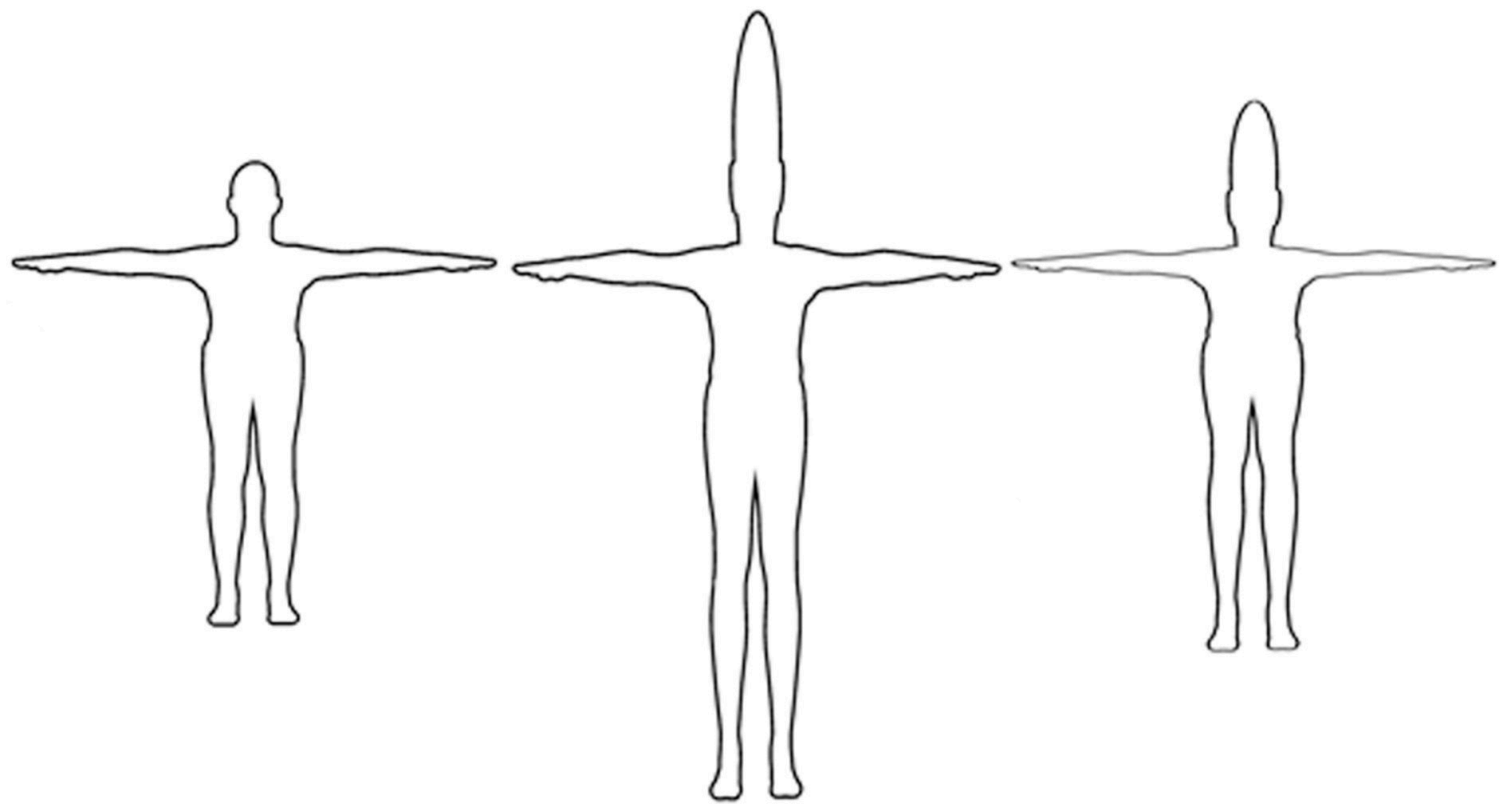
Physical and perceived bodies. Average bodies across participants: **(Left)** physical, based on the measured body heights; **(Middle)** perceived, based on the pointed-out body part locations in the VR headset; **(Right)** perceived, based on the pointed-out body part locations in the Pano-LSID. Scaling is in the vertical dimension only and the arms are not considered in the scaling.

We rescaled the average physical body of the sample of participants, which is based on the average measured body part locations, to average perceived bodies for the sample, based on the average heights pointed at for the different body parts. As the pointing at body parts was performed for the VR headset and for the Pano-LSID separately, we arrived at two perceived bodies, one for the VR headset and one for the Pano-LSID. The results in the form of simple line drawings of these average bodies can be seen in Figure 6. Linkenauger et al. (2015) formed the main inspiration for rescaling the body based on perceived body part locations in this way.

The two rescaled bodies model the same distortions—relative to the physical body (left)— as can be seen in the Figure 5 bar graph, for the VR headset (middle) and the Pano-LSID (right), respectively. What can be seen here as well, are mainly the particularly large overshooting for the top of the head and large undershooting for feet and knees for the VR headset, as well as the large overshooting for the top of the head for the Pano-LSID. Note, that the analyses of self-localization in terms of the perceived body involve individually recomputed body regions and recategorized self-location responses, in contrast to the average rescaled bodies depicted in Figure 6.

### VR Self-Location on the Perceived Body

Since body part localization has large inaccuracies as well as differs per VR setup, for the rest of the analysis of self-location we will use the perceived body per participant. Recomputing the regions of the body in terms of the perceived body and recategorizing the pointing at self-responses in terms of these new body regions, provides a way of looking at the results for self-locating while taking into account how participants perceived their bodies in the two VR setups. Using the recoded responses, the RM-ANOVA was done, in the same way as before (see section VR Self-Location on the Physical Body).

**Figure 7:**
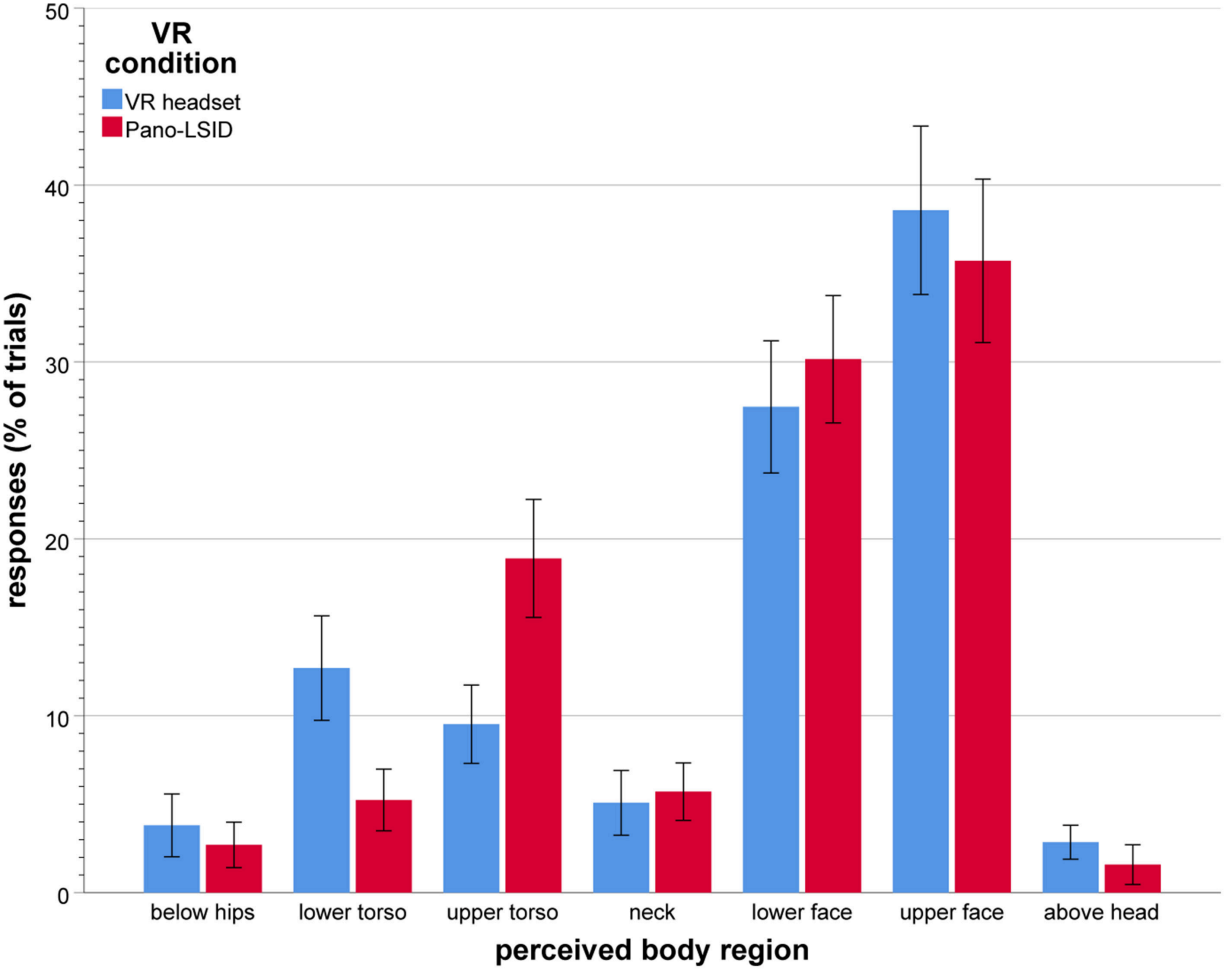
Self-pointing in terms of perceived body regions (in mean percentage of trials per VR setup), by VR setup (*N* = 30; error bars: ± 1 SE).

All results reported here are Greenhouse-Geisser corrected, because of failed Mauchly's tests of sphericity. A significant main effect was found for responses per perceived body region [*F*_(2.37, 68.6)_ = 28.80, *p* < 0.001, η${}_{\text{p}}^{2}$ = 0.50]. There was no significant interaction between body region and VR setup for pointing to self in terms of perceived body regions [*F*_(2.89, 83.7)_ = 2.34, *p* = 0.081, η${}_{\text{p}}^{2}$ = 0.75; see Figure 7 for the responses per perceived body region by VR setup].

Participants did not point to all regions of the body equally, nor did they point to one particular region only. When considering the perceived body, they clearly pointed mostly to the upper face (M = 37.1%, *SD* = 23.2), followed by the lower face (M = 28.8%, *SD* = 14.0), followed by pointing to the upper torso (M = 14.2%, *SD* = 11.8), and then to the lower torso (M = 9.0%, *SD* = 11.3). Hardly any pointing above the head remained after rescaling from physical to perceived body regions. Overall, collapsed over both VR setups, the amounts of pointing remained similar or went up for all body regions after rescaling, except for the neck and below the hips.

Comparisons were made between all body regions (21 comparisons) using the Holm-Bonferroni correction procedure. Holm-Bonferroni corrected paired-samples *t*-tests showed significantly larger amounts of responses for the upper and for the lower face, compared to all other regions, except each other [all *p* < 0.001, except for the comparisons with the upper torso (for the lower face: *p* = 0029; for the upper face: *p* = 0062)]. Significantly larger amounts of responses were also found for the upper torso compared to below the hips (*p* = 0.0023), the neck (*p* = 0.018), and above the head (*p* = 0.00074), as well as for lower torso compared to below the hips (*p* = 0.0496).

Body region is actually not a truly balanced within subject variable and the pointing percentages do not have similar variances across the levels of this variable, nor across the differences between the various levels of body region. Therefore, the self-localization data was analyzed again using a bootstrapping methodology, which does not assume normality of the data. New analysis results were subsequently compared with those from our previous specific comparisons using Holm-Bonferroni corrected paired samples *t*-tests (see paragraph 3 of section VR Self-Location on the Physical Body and paragraph 4 of this section). Bootstrapping versions (resampling 10,000 times) were run of the *t*-tests testing for each physical body region whether there is a significant difference in the mean percentages of trials pointed to it between the two VR conditions (7 comparisons). These alternative bootstrapping *t*-tests yielded similar results as the previous non-bootstrapping *t*-test (section VR Self-Location on the Physical Body) and the same comparisons showed significant differences (for the lower face, the upper face, and above the head). We also ran bootstrapping versions of the *t*-tests comparing all perceived body regions with each other for significant differences in the mean percentages pointed to them (21 comparisons). These alternative bootstrapping *t*-tests also yielded similar results as the previous non-bootstrapping *t*-tests (in this section) and again the same comparisons showed significant differences as before. As the alternative bootstrapping analyses—not making assumptions about the data distribution—yielded very similar results as our previous analyses, they do not call for a change in the interpretation of the data or our conclusions. We therefore believe, that it is also in this case warranted to keep our initial analyses.

The main effects of rescaling the body regions in terms of perceived body are the following. (1) No longer a significant overall effect of VR condition for pointing to self. (2) An increase in pointing to the face, the upper followed by the lower; with almost no pointing for self above the head anymore. (3) As the torso regions also have substantial pointing to them, the results now look more like the bimodal results from the physical setup (Alsmith and Longo, 2014) than after the analysis in terms of regions of the physical body.

### Body Template Task

Overall, pointing height on the body templates as a percentage of total template body height was lower [M = 81.2% (of total template body height), *SD* = 9.3] than pointing height as a percentage of total physical body height in the VR setups (M = 87.5 %, *SD* = 20.5 for the VR headset and M = 88.2, *SD* = 9.3 for the Pano-LSID). In paired-samples *t*-tests, the difference in mean percent pointing height across participants was significantly different for the templates compared to the Pano-LSID [*t*_(29)_ = 3.27, *p* = 0.003, Cohen's d_z_ = 0.61], but not for the templates compared to the VR headset [*t*_(29)_ = 1.48, *p* = 0.145, Cohen's d_z_ = 0.28]. No significant correlations (two-tailed Pearson) were present between the pointing height on the body templates and the pointing height on the physical body in either of the VR setups [templates—VR headset: *r*_(28)_ = −0.073, *p* = 0.700; templates—Pano-LSID: *r*_(28)_ = −0.038, *p* = 0.841). In paired-samples *t*-tests, no significant differences were found between the pointing heights on the different body outlines used in the template task (*p* >> 0.05).

**Figure 8:**
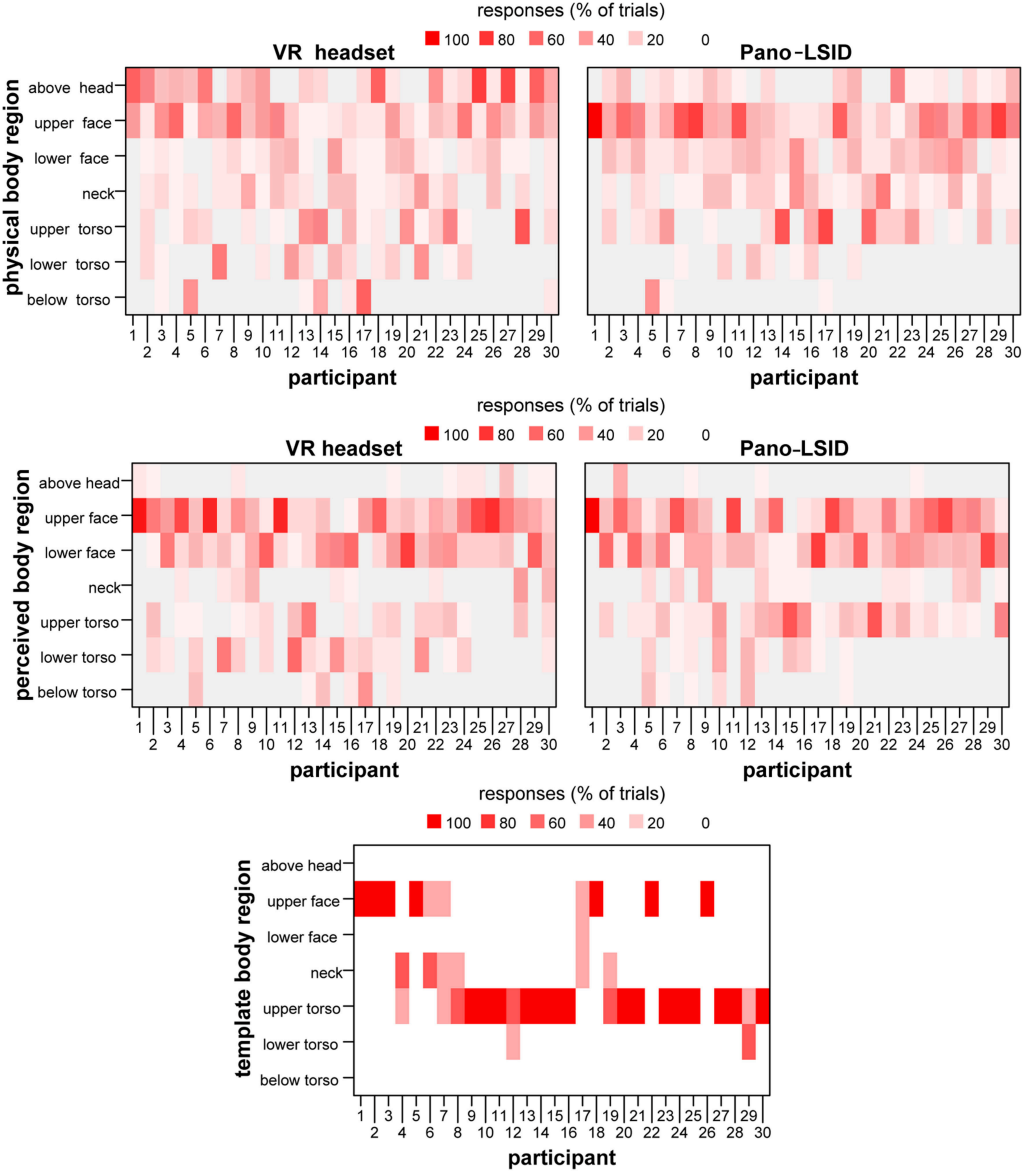
Distribution of pointing at self over the body regions. The percentage of trials pointed at each body region for each participant depicted in a heatmap: **(Upper left)** for regions of the physical body for the VR headset; **(Upper right)** for regions of the physical body for the Pano-LSID; **(Middle left)** for regions of the perceived body for the VR headset; **(Middle right)** for regions of the perceived body for the Pano-LSID; **(Bottom)** for regions of the body templates.

Individual distributions of the responses over the regions of the body are depicted in Figure 8 for the physical body regions, the perceived body regions and regions of the body templates. For the ***physical body***in the VR headset, the largest amount of pointing per individual participant was most frequently above the head (11 of the 30 participants), followed by the upper face (8) and the upper torso (6) (2 to the lower face) (see Figure 8, Upper Left). For the physical body in the Pano-LSID, the largest amount of pointing per individual participant was most frequently to the upper face (19 from the 30 participants), followed by the upper torso (6) and the lower face (4) (2 above the head) (see Figure 8, Upper right). For the ***perceived body***in the VR headset, the largest amount of pointing per individual participant was most frequently to the upper face (13 of the 30 participants) and the lower face (13) (1 to the upper torso and 0 above the head) (see Figure 8, Middle left). For the perceived body in the Pano-LSID, the largest amount of pointing per individual participant was most frequently the upper face (14 from the 30 participants), followed by the lower face (11) (6 to the upper torso and 0 above the head) (see Figure 8, Middle right). For the template bodies, the largest amount of pointing per individual participant was most frequently to the upper torso (20 of the 30 participants), followed by the upper face (9) (see Figure 8, Bottom). Note, that participants can figure more than once in the numbers given in this paragraph on individual distributions of responses over body regions, in case they pointed to more than one region equally, but this was rare.

### Questionnaires

#### Body Perception Questionnaire

On the BPQ, the current sample showed a lower mean score and a higher standard deviation than the norm values for the scale (sample: M = 2.526, *SD* = 0.909; norm: M = 3.026, *SD* = 0.797). Higher interoceptive sensibility was hypothesized to correlate with more accurate body part localization, to be reflected in a negative correlation between BPQ awareness scores and error distances for pointing to body parts. However, no significant two-tailed Pearson correlation was found between BPQ awareness score and absolute error distance for pointing to body parts [*r*_(28)_ = 0.31, *p* = 0.100]. Also no significant two-tailed Pearson correlations were found between BPQ awareness score and pointing height on the body (as a percentage of total physical body height) for any of the self-location tasks [VR headset: *r*_(28)_ = −0.12, *p* = 0.544; Pano-LSID: *r*_(28)_ = 0.027, *p* = 0.889; body templates: *r*_(28)_ = 0.083, *p* = 0.663].

Not finding support for our hypothesis that there might be a correlation between BPQ and pointing performance, might be due to several factors. It could be that the scale we used is actually not very adequate for measuring interoceptive sensibility. It could also be that interoceptive sensibility is not the component of interoception that is most correlated with the ability to locate your body parts [it being a subjective sensibility measure without guarantee of relating to the strength of interoceptive signals, or to objective measures of accuracy on interoception tasks (Garfinkel et al., 2015)]. And it could also be, that there is simply no correlation between interoception and the accuracy in locating body parts in the form hypothesized here. Another option is, that the absence of a significant correlation is somehow related to the current sample having a relatively low mean score on the awareness scale.

#### Post-questionnaire

On the post-questionnaire (see the Supplementary Material), there were three questions concerning the strategies participants had used. On the first, general one (question 3 of the post-questionnaire), eleven of the thirty participants reported to have (on some of the trials) first put the pointer straight at their eyes and then moved it from there to point at other locations, four participants stated that they had imagined a line extending from the pointer to their body and two participants indicated that they had moved the pointer up and down a bit to get a feeling for where it was pointing. Eight participants stated they had used no specific strategy, although most of them did specify one or more on the next two follow-up questions. On the second question (4 in the questionnaire), on pointing to self, eight participants reported that they had pointed at their eyes, seven at their chest, three at their face *or* chest and two at their eyes *or* face. Other body parts were reported, but only once each, and five participants indicated not to have pointed at a specific body part. On the third question (5 in the questionnaire), on pointing to body parts, seventeen participants reported to have felt where their body parts were, two to have imagined a picture of their body, and five to have imagined a line from the pointer to their body. One participant reported feeling *and* imagining a picture and one reported feeling, imagining a picture, *and* imagining a line. Only one participant indicated on this question to have used no strategy. Note, that per question each individual participant only occurs once in the total number of reported cases.

## Summary of Results and Discussion

### Does Pointing to Self and Body Parts Differ Between an LSID and a VR Headset?

For pointing to body parts, there were large differences in accuracy between the VR setups. Namely, while in the Pano-LSID participants were rather accurate with the exception of the top of the head (overshooting) and the feet (undershooting), in the VR headset large errors were found for several body parts, especially the feet, knees, and hips (undershooting) and top of the head (overshooting). Also, the VR-headset had overall undershooting, while the Pano-LSID had overall overshooting of body part locations. Our expectation was therefore supported for body parts: there were significant differences between the VR setups in the error distances for several body part locations.

That pointing to body parts was found to be not fully accurate and to differ between VR setups may be due to the following reasons. Not having visual access to one's own body in the VR headset likely makes both body part and body boundary localizing more difficult. Moreover, having visual access to your body part locations might also improve the accuracy for locating non-visible body parts, such as the top of your head; or it may enhance general body awareness. Not having visual access may therefore partially explain the less accurate pointing to body parts in general, as well as the larger undershooting for the feet, knees, and the hips and the larger overshooting for the top of the head in the VR headset. Finally, instead of promoting pointing to the face, the headset may also have decreased the amount of pointing to the face. This may have resulted from the headset forming a barrier between the pointer and the participant, or from it being experienced as a strange object that is not you.

Egocentric distance perception is known to be inaccurate in both VR headsets and in LSIDs. However, distortions in distance perception, and thereby in the perceived distance of the pointer, may have been larger in the VR headset than in the Pano-LSID in the current study (Piryankova et al., 2013; Young et al., 2014; Creem-Regehr et al., 2015) and this may partially explain the differences in the findings for the two VR setups. Moreover, we chose a distance to the pointer stimuli known to minimize misperception in the Pano-LSID (3.5 m; Piryankova et al., 2013) and used the same distance in the VR headset. If the pointer in the current VR setups appeared closer than it was, this may on average have resulted in extremer pointing angles than actually needed to point to specific bodily locations. This may also partially explain the larger amount of overshooting above the head for self-location for the VR headset compared to the Pano-LSID, as well as the larger amounts of undershooting reflected in pointing below the hips and to the lower torso.

Self-pointing based on the physical body was found to differ between VR setups. Since perceived body part locations also differed between VR setups, it became important to use perceived body regions to see whether the differences in self-pointing were due to differences in body part localization. Overall, after rescaling the self-pointing to the perceived body, there was no interaction between body region and VR condition for self-pointing. Differences in physical vs. perceived body part locations were therefore likely the reason self-location differed between VR setups when considering physical body regions. Our expectation was thus not supported for self-localization: there was no significant difference in self-localization between the VR setups used, when body part localization in each VR setup was taken into account.

After rescaling to the perceived body, the current results look more like the bimodal self-pointing (to the upper torso and the upper face) found previously in a physical setup (Alsmith and Longo, 2014). The results are also somewhat consistent with the predominant pointing to the upper face as found in Van der Veer et al. (2018) where no rescaling to perceived body regions was performed.

This effect of rescaling to the perceived body suggests that, taking into account where people estimate their body parts to be as well as the differential distortions in spatial perception between VR setups, makes it possible to better understand where people locate themselves in VR. Distortions in body perception in VR may therefore be confined to distortions in the localization of body parts, rather than also involving where people ultimately locate themselves as such.

### Is Indicated Self-Location in the Body Template Task Outside of VR Similar to Self-Localization in VR?

On the body template task, the mean pointing height (in percentage of total template body height) was lower than the mean pointing height (in percentage of the participants' total **physical** body height) in both VR setups (significantly lower compared to the Pano-LSID only, not compared to the VR headset). In contrast to VR self-pointing, mean pointing per participant in the body template task was most frequently to the upper torso, followed by the upper face. No correlation was found between mean pointing height on the body in the template tasks and mean pointing height on the physical body in either VR setup. These findings confirm our expectations and are in line with those of Van der Veer et al. (2018), but not with the other earlier experiments employing outlines of human bodies as discussed in the introduction (Limanowski and Hecht, 2011; Starmans and Bloom, 2011; Anglin, 2014). These experiments all found the location of the self to be indicated (most often) in the face or related areas, such as the brain or eyes, rather than in the torso.

The difference in our findings for the template task compared to VR could be due to several factors: the perspective on the body; the potentially lower identification with the body outlines as compared to one's own body; and possibly a general tendency to point to the center of an object (or, more precisely, on the medial axis skeleton: Firestone and Scholl, 2014). Moreover, in a perspective-taking study it has been found that people value their own minds more than their bodies, but often fail to realize that others do so as well, assuming others value their own bodies more than their minds (Jordan et al., 2019). As pointing on the template may resemble pointing to someone else (counter to task instructions), this bias may also have promoted the smaller amount of pointing on the template to the (upper) face, where typically the mind is thought to reside. Our findings are nicely in line with Alsmith et al.'s (2017) findings of self-location being distributed between head and torso, with larger contributions for the torso. In this study, self-location was implicated by the part(s) of the body used by participants to indicate the locations of external objects relative to themselves. Thereby it shares with our template task that the performed action was not directed at one's own physical body.

### Where do People Precisely Locate Themselves in Their Bodies?

In two VR setups we found that when asked to point directly at themselves, overall participants pointed to the face most (upper followed by lower), followed by the torso regions and with some pointing to all regions of the body, as well as above the head. This is largely consistent with previous VR findings (Van der Veer et al., 2018), showing predominantly pointing to the upper face in a VR headset, and Alsmith and Longo's (2014) results from a wholly physical setup, where bimodal pointing to the upper torso and upper face was found.

For self-pointing, the predominant pointing to the upper face found in Van der Veer et al. (2018) was interpreted as possibly resulting from wearing a VR headset, i.e., from drawing more attention to the face/eyes (resulting from the pressure and weight of the headset) and from not having visual access to one's own body. However, in the present study, we also found a large amount of pointing to the upper face in the Pano-LSID—where no special emphasis was placed on the head and participants had visual access to their bodies. Interestingly, the slightly larger amount of pointing to the (upper) torso for the self-location pointing in the Pano-LSID as compared to the VR headset is more similar to outside of VR (Alsmith and Longo, 2014)—with which it has visual access to the body in common. Therefore, it seems that people mostly do localize themselves in the face and the torso. This idea gets some further support from the locations participants reported to have pointed to, as stated on the post-questionnaire. For self-locating, out of the 30 participants 10 reported to have pointed to the eyes or face, seven to the chest (upper torso) and three to both the face and the chest.

## General Discussion

The main finding of this study is that the VR setup influences where people point to their body parts, but not to themselves (as long as you take into consideration the perceived body part locations). In particular, we found large differences in body part localization between a VR headset and an LSID. For body parts, we found distortions in body perception as described in non-VR setups [e.g., Linkenauger et al. (2015) and Fuentes et al. (2013)]. Additionally, we found that estimations of the boundaries of the body seem to be heavily distorted in a VR headset and to a lesser extent in an LSID. Body part localization likely differs in these two setups due to at least two factors, differences in distance estimation and differences in access to the visual body. In a follow-up version of the VR headset condition, it could therefore be interesting to connect to current technological possibilities further and to provide the participants with visual cues about their bodies in the form of (partial) (tracked) self-avatars. When switching to a more recent addition to the VR headset hardware market for easier implementation of body tracking, the issue of distance underestimation may be further reduced as well.

When rescaling self-pointing to the perceived body, participants point mostly to the face, as well as to a lesser extent to the torso. Finally, pointing in VR differs from the body template task where pointing to self was found to be primarily to the upper torso. Our results suggest that experimental paradigms using VR as a tool to study aspects of the bodily self and body perception should consider that the technology itself may influence body part localization, and also self-location estimates if inaccuracies in body part localization in the specific VR setup are not taken into consideration.

The implication for the use of virtual reality technology is primarily that users may be uncertain of where exactly their body parts (especially their bodies' boundaries) are in the virtual world. This implication clearly relates to several of the fundamental challenges for virtual environments as described by Slater (2014). First, since VR is likely moving to the home and being used by large numbers of people, there is a need to understand how VR might influence body part localization and awareness, especially after longer exposure times. Since how you perceive and how you act are tightly connected, it is clear that a different perception of one's own body in VR may result in acting differently in VR. Finally, as Slater points out, virtual reality profits from exploitation of the brain to produce illusions of perception and action. Our results suggest that fundamental properties of body perception can be altered depending on the technology used. In order to exploit these illusions properly, a more complete understanding of the baseline of how human perception works in VR may be needed. Fortunately, pointing to self remains unchanged in the two current VR setups when considering the perceived body, based on body part localizations. This suggests that the sense of self-location is consistent across vastly different VR technologies and is primarily in the face and torso regions.

## Ethics Statement

All participants gave written informed consent. Procedures were in accordance with the principles of the Declaration of Helsinki and approved by the Ethics Committee of the University Hospital Tübingen.

## Author Contributions

AvdV: conceptualization, analysis, investigation, methodology, visualization, writing: original draft, writing: review and editing. ML: conceptualization, methodology, writing: review and editing. AA: conceptualization, methodology, writing: review and editing. HW: conceptualization, writing: review and editing. BM: conceptualization, analysis, methodology, visualization, writing: original draft, writing: review and editing.

### Conflict of Interest Statement

BM is a review editor for Frontiers in Robotics and AI: Virtual environments. The remaining authors declare that the research was conducted in the absence of any commercial or financial relationships that could be construed as a potential conflict of interest.

## Supplementary Material

Table S1 The merged data from all VR pointing tasks from all participants.

Table S2 The data from the pointing at the body templates for all participants.

Data Sheet 1 Post-questionnaire.

## Abbreviations

AN: anorexia nervosa
BIT: body image task
BPQ, Body perception questionnaire (Porges: 1993)
FOV: field of view
LSID: large-screen immersive display
Pano-LSID: panoramic large-screen immersive display
RM-ANOVA: repeated-measures analysis of variance
VR: virtual reality.
