# Supplementary material for: Self and Body Part Localization in Virtual Reality: Comparing a Headset and a Large-Screen Immersive Display
Source: Front Robot AI. 2019 May 8;6:33. doi: 10.3389/frobt.2019.00033 (PMC7805778; doi:10.3389/frobt.2019.00033)
Supplement: Data Sheet 1 — Post-questionnaire. [file Data_Sheet_1.PDF]

## Post-questionnaire

**Please circle the relevant answers, give number ratings where asked, and fill out the open questions.**

Participant number: ___________________________________________

Date: ________________________________________________________

Time: ________________________________________________________

1. Did you understand the tasks?   yes   no
2. Were the tasks clear and easy to understand?   yes   no

If not, what was unclear or difficult? ______________________________________________________

_____________________________________________________________________________________

_____________________________________________________________________________________

3. Did you use a specific strategy for deciding where to direct the pointer to?   yes   no

If so, what did you do? _________________________________________________________________

_____________________________________________________________________________________

_____________________________________________________________________________________

4. When pointing directly at you, did you point at a specific body part?  yes   no

If so, which one? ____________________________________________________________________

5. When pointing directly at body parts, did you use one of the following specific strategies?

Feeling where your body parts are?   Imagining a picture of your body?

Other? ______________________________________________________________________________

Don't know.

6. Can you indicate how far away from you the pointer was located (in centimetres)?

On the large screen: ____            In the head-mounted display: ____

7. Over the course of the experiment did you …

… become tired? ______________________________________________________________________

… lose interest? ______________________________________________________________________

8. Did you feel some tasks within one (or more) part(s) of the study were more difficult than others in the same part?   yes   no

If so, which were more difficult? ________________________________________________________

_____________________________________________________________________________________

_____________________________________________________________________________________

9. Can you rate the confidence you have in your responses (1 (lowest) – 100 (highest)), for each part of the experiment (not necessarily listed in the order you have done them)?

a. Pointing at self, head-mounted display: ____ b. Pointing at self, large screen: ____

c. Pointing at body parts, head-mounted display: ____ d. Pointing at body parts, large screen: ____

10. Do you have any ideas on what the research questions may be? ________________________

________________________________________________________________________________

________________________________________________________________________________

11. Do you have anything else on your mind related to the experiment? ____________________

________________________________________________________________________________

________________________________________________________________________________

12. What is your age? ___________

13. What is your gender? ___________

14. What is your handedness? right left

15. Do you play sport? yes no

If so, how many hours per week on average? ___________

16. Do you do yoga, Pilates, or something similar? yes no

If so, how many hours per week on average? ___________

17. Do you meditate? yes no

If so, how many hours per week on average? ___________

18. What is your profession/occupation? ___________

19. What percentage of your waking hours do you on average spend

- seated: ___________
- standing: ___________
- walking: ___________
- doing physical labour: ___________

20. At what time did you eat your last meal? ___________

21. How many hours ago did you eat your last meal? ___________

22. Are you religious? yes no

If so, how many hours per week do you perform specific religious practice on average? ___________

23. What is your nationality? ___________

24. In which country(-ies) did you grow up? ___________

25. Do you have children? yes no

26. What is the highest level of education you have finished? ___________

27. Do you currently have any pain? yes no

If so, where are you experiencing pain? ___________

28. Have you experienced virtual reality before? yes no

If so, how many hours in total? ___________

29. Do you play video games? yes no

If so, how many hours per week on average? ___________

30. Did you experience any unpleasant feelings during the experience? yes no

If so, what were they? ___________

31. What do you think was the purpose of the experiment? ______________________________

______________________________________________________________________________________

______________________________________________________________________________________

32. Do you wear glasses? yes no 33. If so, today? yes no

32. Do you wear lenses? yes no 35. If so, today? yes no

1. Please rate your self-confidence (1 (lowest) – 100 (highest)): ___________
2. Please rate your overall stress level (1-100): ___________
3. Please rate your overall happiness (1-100): ___________
4. Please rate yourself on an introversion-extraversion scale (1 (most introvert) – 100 (most extravert)): ___________
5. Please rate how healthy you currently feel (1-100): ___________
6. Please rate how rested you currently feel (1-100): ___________
7. How many hours have you slept last night? ___________
8. Please rate how energetic you currently feel (1-100): ___________
9. Please rate how much you enjoyed the experiments (1-100): ___________
10. To what extent did you feel you were doing your best (1-100)? ___________
11. To what extent did the experiments hold your attention (1-100)? ___________
12. To what extent were you focused on the experiments (1-100)? ___________
13. To what extent were you aware of yourself in your environment (1-100)? ___________
14. To what extent were you aware of your body (1-100)? ___________
15. To what extent did you want to stop the experiment (1-100)? ___________
16. To what extent did you feel separated from your real-world environment (1-100)? ________
